# Loneliness, Social Contact, and Health Among Older Adults During the COVID-19 Pandemic

**DOI:** 10.1093/geroni/igab046.502

**Published:** 2021-12-17

**Authors:** Soyoung Choun, Carolyn Aldwin, Dylan Lee

**Affiliations:** Oregon State University, Corvallis, Oregon, United States

## Abstract

The COVID-19 pandemic is a challenging situation for many older adults at elevated risk for mortality. Social distancing and lockdown to prevent contagion may result in social isolation and feelings of loneliness, which can have adverse effects on health. We examined how depressive symptoms were associated with between-person differences and within-person variations in loneliness, social contacts, and daily physical problems during 8 weeks. We sampled 247 older adults (Mage = 71.1, SD = 7.3, range = 51 - 95), who participated at micro-longitudinal online surveys (baseline and 7 weekly follow-ups) from April 28 to June 23. Multilevel modeling analysis controlling age, gender, marital status, and education showed that depressive symptoms were significantly decreased during 8 weeks. Further, depressive symptoms were positively coupled with both loneliness and physical problems for both the within-and between-person levels. Increase in social contact was related to decreases in depressive symptoms only at the between-person level.

